# Identification and functional prediction of lncRNAs associated with intramuscular lipid deposition in Guangling donkeys

**DOI:** 10.3389/fvets.2024.1410109

**Published:** 2024-07-05

**Authors:** Yongdong Peng, Mingzhu Zhu, Yunyi Gong, Changfa Wang

**Affiliations:** Liaocheng Research Institute of Donkey High-Efficiency Breeding and Ecological Feeding, Agricultural Science and Engineering School, Liaocheng University, Liaocheng, China

**Keywords:** Guangling donkey, lncRNA, longissimus dorsi muscle, lipid deposition, regulate

## Abstract

Many studies have shown that long non-coding RNAs (lncRNAs) play key regulatory roles in various biological processes. However, the importance and molecular regulatory mechanisms of lncRNAs in donkey intramuscular fat deposition remain to be further investigated. In this study, we used published transcriptomic data from the longissimus dorsi muscle of Guangling donkeys to identify lncRNAs and obtained 196 novel lncRNAs. Compared with the coding genes, the novel lncRNAs and the known lncRNAs exhibited some typical features, such as shorter transcript length and smaller exons. A total of 272 coding genes and 52 lncRNAs were differentially expressed between the longissimus dorsi muscles of the low-fat and high-fat groups. The differentially expressed genes were found to be involved in various biological processes related to lipid metabolism. The potential target genes of differentially expressed lncRNAs were predicted by cis and trans. Functional analysis of lncRNA targets showed that some lncRNAs may act on potential target genes involved in lipid metabolism processes and regulate lipid deposition in the longissimus dorsi muscle. This study provides valuable information for further investigation of the molecular mechanisms of lipid deposition traits in donkeys, which may improve meat traits and facilitate the selection process of donkeys in future breeding.

## 1 Introduction

Long non-coding RNA (lncRNA) is a class of non-coding RNAs with a length greater than 200 nucleotides. Increasing evidence suggests that lncRNA plays important roles in various biological processes, such as embryonic development (1, 2), gene expression regulation (3, 4), reprogramming (5, 6), and genomic imprinting (7, 8). Additionally, many lncRNAs have been found to be involved in regulating lipid metabolism. For example, lncRNA IMFNCR promotes chicken myoblast differentiation by sequestering miR-128-3p and miR-27b-3p (9), lncRNA 332,443 inhibits preadipocyte differentiation by targeting Runx1, p38 MAPK, and ERK1/2-MAPK signaling pathways (10), and LncLSTR forms a molecular complex with TDP-43 to regulate the expression level of Cyp8b1, thereby affecting the FXR regulatory pathway, leading to increased apoC2 levels and influencing triglyceride levels. LNCLSR directly binds to TDP-43 to inhibit Cyp8b1 expression and subsequently regulate triglyceride levels (11).

Guangling donkeys are distributed in Guangling County, Shanxi Province, China, and they are a local dominant breed that is carefully reared by local people using traditional production practices (12). Guangling donkeys have a stout physique and full muscles. Guangling donkeys used for meat production have a high intramuscular fat (IMF) content; however, the underlying molecular mechanisms underlying the IMF variation among donkey species are not fully understood.

IMF, also known as marbling, is an important indicator of the lean meat-to-fat ratio, which directly affects the tenderness, juiciness, and flavor of the meat (13). IMF content is one of the most important indicators used to evaluate meat quality (14). IMF content is a polygenic trait that is regulated by many genes affecting adipogenesis and lipid metabolism (15). At present, the underlying molecular variations affecting IMF content among donkey breeds are unclear.

This study used published transcriptome data from the longissimus dorsi muscle of a Guangling donkey to identify lncRNA and conducted differential expression studies of coding genes and lncRNA, constructing an expression regulation network, which lays the foundation for further analyzing the molecular mechanisms of lipid deposition traits in donkeys.

## 2 Materials and methods

### 2.1 Data sources

A total of 30 Guangling donkeys were raised on a commercial donkey farm in Fanshi County, Xinzhou City, Shanxi Province, China, and 6 donkeys with IMF differences and similar ages were selected (age: 2–3 years old, weight: 232–245 kg; female) for use in this study. All Guangling donkeys were reared under the same natural conditions of uncontrolled room temperature and light with unrestricted access to food and water. The longissimus dorsi samples at the 13th rib were aseptically and quickly obtained within 30 min of harvest. The collected samples were stored in liquid nitrogen for immediate storage, and long-term storage was carried out at −80°C. According to the China National Standard GB5009.6-2016 “Determination of Fat in Foods in National Food Safety Standard,” the IMF content was determined by the Soxhlet method. A Soxhlet extraction apparatus was used to remove fat and dry the ground meat samples for fat extraction. Petroleum ether was used as a solvent. This was recycled and dried for 8 h. Then, it was weighed to obtain the weight of the bottle containing fat. The IMF content was calculated by a formula. The three longissimus dorsi samples with the highest IMF contents and the other three with the lowest IMF contents were selected for transcriptome analysis (12). A total of six RNA-seq datasets were obtained from a previously published study and downloaded from the NCBI’s GEO database (PRJNA658642) (12). The donkey gene annotations were downloaded from https://ftp.ensembl.org/pub/release-110/gtf/equus_asinus. Moreover, the Non-Redundant Protein Sequence (NR) Database was downloaded from ftp://ftp.ncbi.nih.gov/blast/db/. The uniref90 database was downloaded from https://ftp.uniprot.org/pub/databases/uniprot/uniref/uniref90.

### 2.2 RNA-seq reads mapping and transcriptome assembly

The quality of sequencing reads was evaluated by FastQC command. The raw reads were filtered and trimmed by Trimmomatic (version 0.39) with default parameters (16). The clean reads were then mapped to the donkey reference genome [Ensembl: ASM1607732v2 (GCA_016077325.2)] by HISAT2 v2.2.1 with the default parameters (17–20). StringTie (version 2.2.1) was used to assemble the mapped reads with default parameters (17). Then, the merge tool of StringTie was used to merge the six assembled transcript files (GTF format) of the two groups into a non-redundant transcriptome. In addition, by using the assembled GTF file, StringTie software was used to estimate the expression levels of genes and transcripts in all samples for subsequent studies with the parameters “-e” and “-B” (17, 18, 21).

### 2.3 LincRNAs identification pipeline

**Figure 1:**
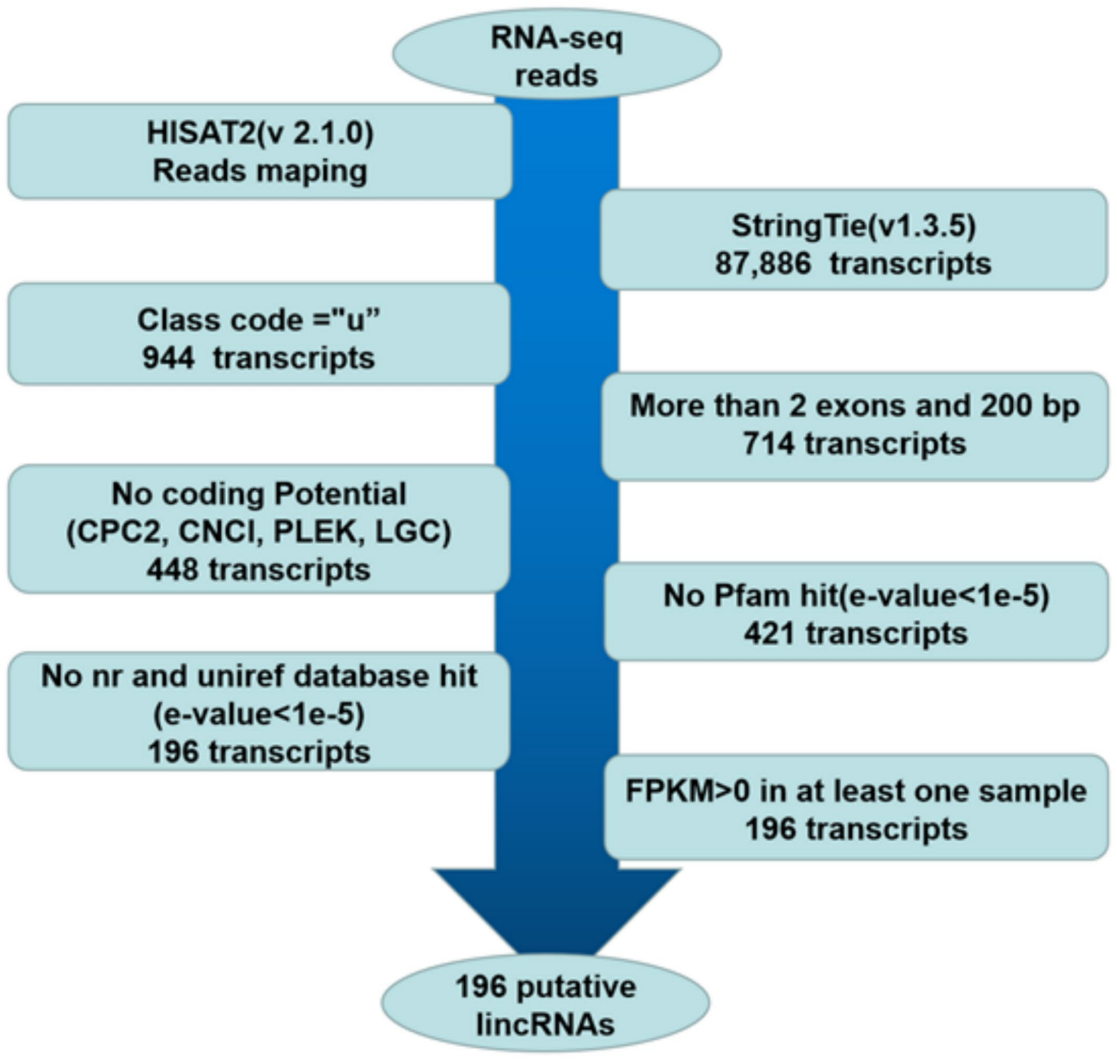
Pipeline for the identification of putative lincRNAs in this study. The frames in the direction of the arrow show the filtering process and the number of screened transcripts. “u”: Unknown, intergenic transcript; CPC2: coding potential calculator 2; nr: non-redundant protein sequence database; FPKM: fragments per kilobase of transcript per million mapped reads.

The pipeline for lincRNA (long intergenic non-coding RNA) identification was as follows (Figure 1): (1) retained those transcripts with “u” category categorized by using gffcompare, which indicated intergenic transcripts (20, 21). (2) According to the merged GTF file, the transcripts with single exons and less than 200 bp in length were removed (18, 21). (3) The CPC2, CNCI, PLEK and LGC were used to assess the protein-coding potential of complete transcript sequences, and the transcripts that cannot encode proteins based on protein-coding potential were retained (22). (4) The HMMER was used to identify the transcripts translated in all six possible frames with homologs that were concluded in any of the known protein family domains in the Pfam database, and transcripts that matched to the Pfam hit (*E*-value < 1e-5) were excluded (18–21, 23). (5) BLASTX program (24) was used to filter out any transcripts that have similarities to known proteins in the NCBI NR and UniRef90 databases (*E*-value < 1e-5) (20, 21). (6) Reserve transcripts with FPKM values greater than 0 in at least one sample (18–20).

### 2.4 Comparisons between lncRNAs and protein-coding transcripts

We selected the transcripts annotated as “protein-coding” in the gene annotation file, and the obtained lncRNAs were screened with “known” and “novel” by “blastn” command. The transcript length, exon length, and exon number of lncRNAs were compared with those of protein-coding transcripts (20).

### 2.5 Analysis of differentially expressed genes and differentially expressed lncRNAs

DESeq2 tool was used to perform differential expression analysis of protein-coding genes and lncRNAs between the high (H) fat group and low (L) fat group (25). |log2 fold change | ≥ 1 and adjusted *p*-value (padj) < 0.05 were used to screen differentially expressed genes (DEGs) and lncRNAs (26).

### 2.6 Prediction of potential target genes

We predicted the molecular functions of protein-coding genes regulated by RNA in cis and trans. First, the neighboring protein-coding genes near DELs (<100 kb) were identified based on cis-prediction principles using Bedtools (18–20, 27, 28). For the trans-regulation of DELs, we calculated the Pearson’s correlation coefficient (r) between DELs and protein-coding genes. We selected protein-coding genes with a Pearson’s correlation coefficient |*r*| ≥ 0.95, *p*-value ≤ 0.01 as potential target genes (PTGs) of DELs (21, 29).

### 2.7 Functional enrichment analysis

Gene Ontology (GO) enrichment analysis was performed by clusterProfiler (30, 31). KOBAS v3.0^1^ was used for the Kyoto Encyclopedia of Genes and Genomes (KEGG) pathway enrichment analysis (32). A *p*-value of less than 0.05 was considered statistically significant (33).

## 3 Results

### 3.1 Summary of RNA-seq data mapping and transcripts assembly in longissimus dorsi muscles

RNA-seq data involving two groups of Guangling donkeys were obtained from a previously published study (12). The six longissimus dorsi muscles (three in each group) with the lowest and highest IMF contents were named the L group and H group, respectively. The individual samples in the groups were named L1, L2, L3, H1, H2, and H3. The clean reads were mapped to the donkey reference genome using HISAT2. Approximately 86.02–90.51% of clean reads from each library were mapped to the donkey reference genome, and 79.76–84.78% of the reads were uniquely mapped to the genome. Then, the transcriptome was assembled for each library by StringTie, and all transcripts were synthesized into non-redundant transcripts using StringTie-Merge. After merging non-redundant transcripts, approximately 1.07% (944 of 87,886) of the transcripts were intergenic transcripts. The 196 putative lincRNAs were obtained according to the illustration shown in Figure 1 (Supplementary Table S1).

### 3.2 Comparison of coding genes and lncRNA features

Previous studies showed that there are many differences between protein-coding transcripts and lncRNAs (18–20, 27, 34–36). According to the assembled transcriptome, the characteristics of lncRNA and protein-coding transcripts were compared. A total of 51,112 protein-coding transcripts, corresponding to 20,553 protein-coding genes annotated in donkeys, were acquired. In addition, the donkey annotation file contains 7,615 known lncRNA transcripts that correspond to 4,709 lncRNA genes (Supplementary Table S1).

**Figure 2:**
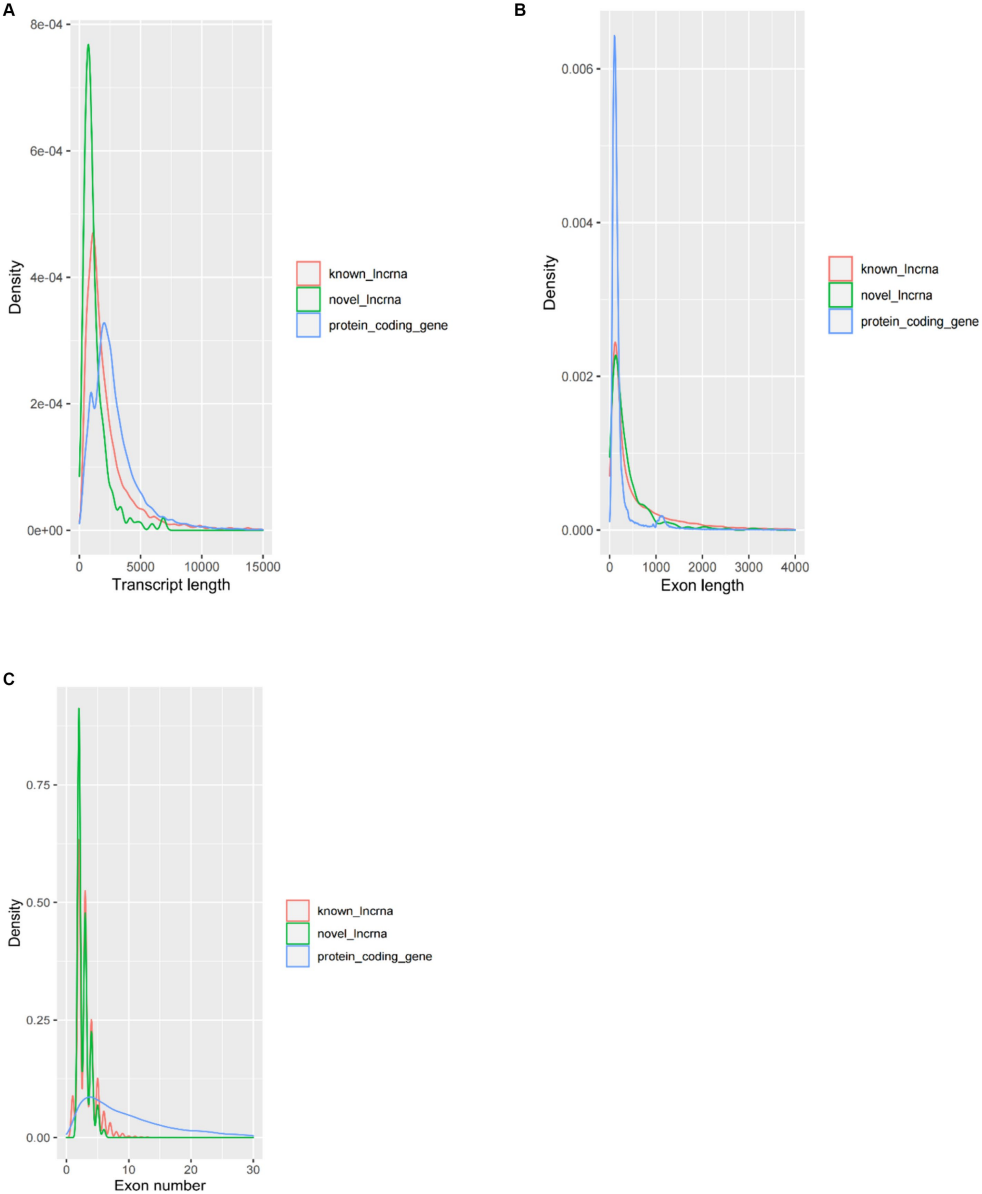
Comparison of the characteristics of protein-coding genes and lncRNA genes. **(A)** Comparison of transcript length, **(B)** comparison of exon length, and **(C)** comparison of exon number.

The average transcript length of the protein-coding transcripts (2,919 bp) was longer than the novel lncRNA transcripts (1,217 bp) and the known lncRNA transcripts (2,394 bp) (Figure 2A). In terms of average exon length, the novel lncRNA is 448 bp in length, shorter than the known lncRNA gene (766 bp) but longer than the protein-coding transcripts (271 bp) (Figure 2B). In addition, we found that the average number of exons in the protein-coding transcripts is 10.7, which is significantly higher than that of the novel lncRNA (2.7) and the known lncRNA (3.1) (Figure 2C).

### 3.3 Differential expression analysis of coding genes and lncRNAs

**Figure 3:**
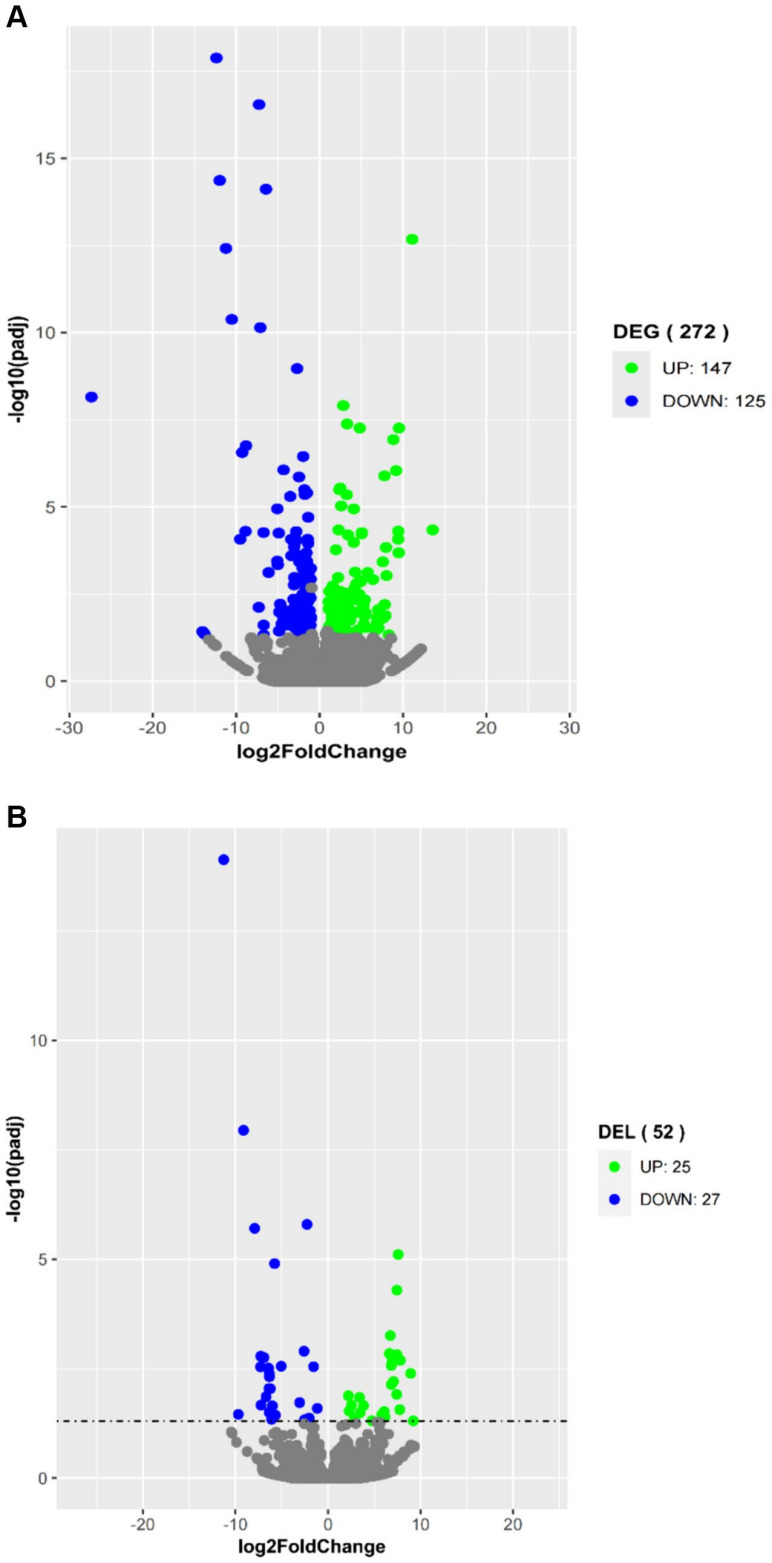
Volcano plots in analyzing differentially expressed genes (DEGs) **(A)** and differentially expressed lncRNAs (DELs) **(B)** in longissimus dorsi muscles with high and low intramuscular fat contents. The green plot represents upregulated expression in the high group; the blue plot represents downregulated expression in the high group; and the gray plot represents no significance.

To explore their potential biological functions, we performed differential expression analysis of coding genes and lncRNAs. A total of 272 DEGs were obtained by comparing the high-fat group with the low-fat group of the longissimus dorsi muscle samples. Among them, 147 genes were upregulated in the high-fat group, and 125 genes were downregulated (Figure 3A). Additionally, 52 differentially expressed lncRNAs (DELs) were identified, with 25 lncRNAs upregulated and 27 lncRNAs downregulated in the high-fat group compared to the low-fat group (Figure 3B).

### 3.4 Functional analysis of differentially expressed genes

To investigate the function of DEGs, we performed GO and KEGG analyses, respectively. GO analysis of DEGs showed that integrin-mediated signaling pathway, cellular response to lipopolysaccharide, trachea development, cellular response to molecule of bacterial origin, and positive regulation of myeloid leukocyte-mediated immunity were the most abundant terms in the biological process category. In terms of cellular component category, actin cytoskeleton, phagocytic cup, cell leading edge, TORC2 complex, and basolateral plasma membrane were the top five terms, while rRNA binding, pattern recognition receptor activity, motor activity, protein kinase binding, and ion channel binding were most prevalent in the molecular function (Supplementary Table S2). Some GO terms were significantly associated with lipid metabolisms, such as response to lipids, cellular response to lipids, glycerophospholipid metabolic process, glycerolipid metabolic process, unsaturated fatty acid biosynthetic process, and phospholipid dephosphorylation. KEGG analysis indicated that DEGs were significantly enriched in 62 KEGG pathways, of which several pathways were related to lipid metabolism, such as the Sphingolipid signaling pathway and MAPK signaling pathway. In addition, some other pathways are related to lipid metabolism, namely fatty acid metabolism, glycerophospholipid metabolism, biosynthesis of unsaturated fatty acids, PI3K-Akt signaling pathway, fatty acid degradation, ether lipid metabolism, cholesterol metabolism, PPAR signaling pathway, TGF-beta signaling pathway, and Wnt signaling pathway (Supplementary Table S3).

### 3.5 Prediction and functional analysis of lncRNA target genes

Many studies have indicated that lncRNA may regulate adjacent genes in a *cis* manner (18–20, 29, 37–41). For PTGs regulated by lncRNAs in cis (<100 kb), we identified a total of 323 PTGs, corresponding to 52 DELs (Supplementary Table S4). To explore the function of putative lncRNAs, GO and KEGG analyses were performed on expressed protein-coding genes transcribed near lncRNA (<100 kb). The results indicated that 102 of 323 PTGs were significantly involved in 143 biological processes (Supplementary Table S5). Of them, 59 PTGs significantly participated in 32 pathways (Supplementary Table S6), including 2 pathways related to lipid metabolism, such as biosynthesis of unsaturated fatty acids and fatty acid metabolism.

**Figure 4:**
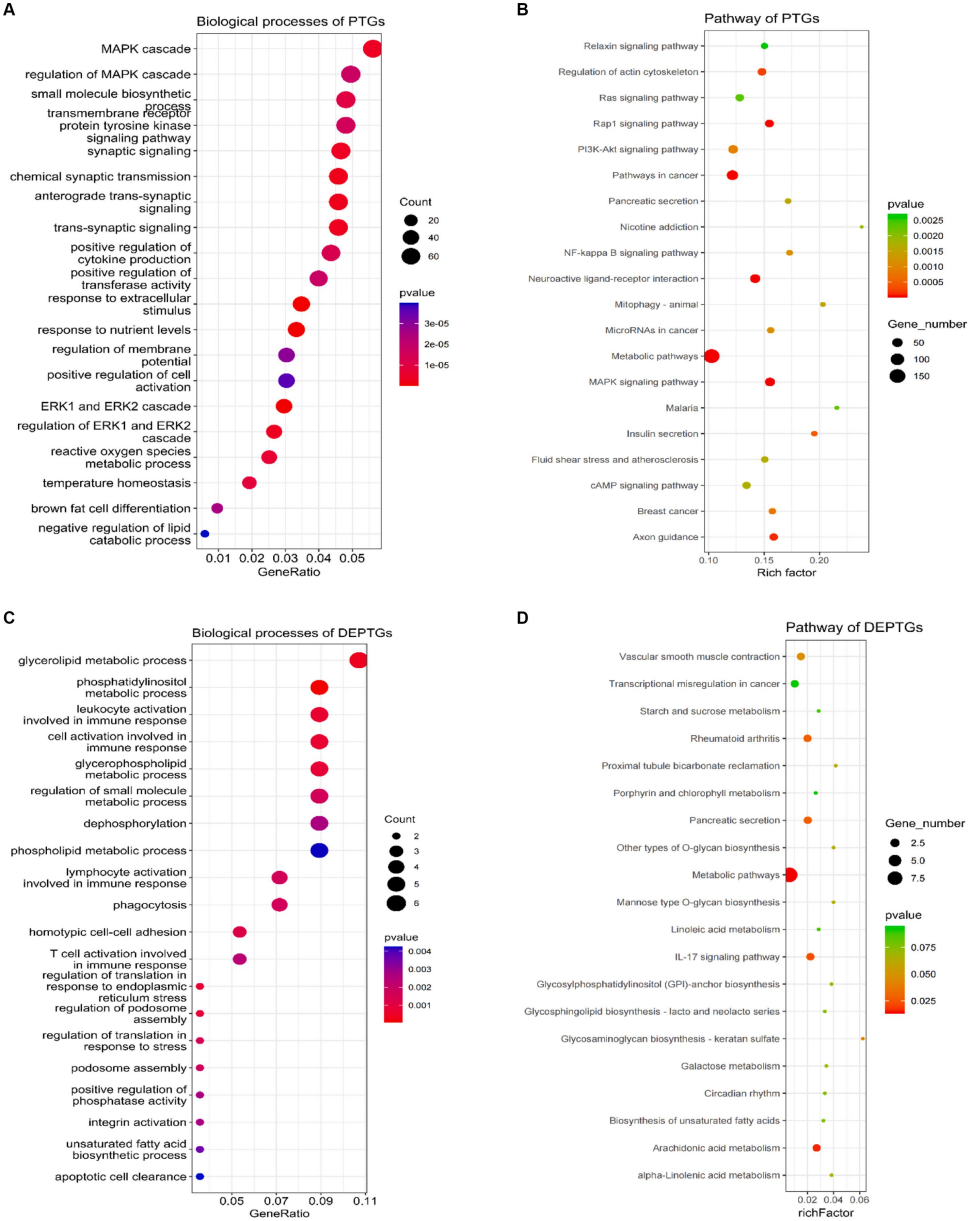
Gene Ontology and pathway analysis of PTGs of DELs. **(A)** Biological processes analysis of PTGs of DELs, **(B)** pathway analysis of PTGs of DELs, **(C)** biological processes analysis of DEPTGs of DELs, and **(D)** pathway analysis of DEPTGs of DELs. PTGs, potential target genes; DELs, differentially expressed lncRNAs; DEPTGs, differentially expressed potential target genes.

LncRNAs can not only regulate the expression of neighboring protein-coding genes through a cis mechanism but also regulate the expression of genes located on other chromosomes via a trans mechanism (42). In this study, we carried out the trans analysis to find the PTGs that were significantly correlated (|*r*| ≥ 0.95, *p* ≤ 0.01) to the DELs. In total, 3,366 PTGs were highly correlated with 52 DELs. Among these genes, 132 PTGs were differentially expressed in groups as DEPTGs, suggesting that most of the lncRNAs regulated gene expression through trans regulation. GO enrichment analysis showed that 3,366 PTGs were enriched in 835 biological processes and 132 DEPTGs were enriched in 143 biological processes (Supplementary Tables S7, S8). In cases of biological process, some GO terms were significantly associated with lipid metabolism, such as negative regulation of the lipid catabolic process, regulation of the lipid metabolic process, negative regulation of the lipid metabolic process, regulation of the lipid biosynthetic process, cellular lipid catabolic process, negative regulation of the lipid biosynthetic process, regulation of fatty acid transport and regulation of the lipid catabolic process (Figures 4A,C). In addition, 3,366 PTGs and 132 DEPTGs were enriched in 74 pathways and 7 pathways, respectively (Supplementary Tables S9, S10). KEGG pathways were involved in the MAPK signaling pathway, PI3K-Akt signaling pathway, glycerolipid metabolism, ether lipid metabolism, fat digestion, and absorption (Figures 4B,D). The results indicated that DELs had an important role in regulating their PTGs that regulate lipid metabolism in longissimus dorsi muscles.

## 4 Discussion

In the present study, transcriptome sequencing of longissimus dorsi tissues with different IMF contents from Guangling donkeys was used to investigate genes and lncRNA related to lipid metabolism in the longissimus dorsi muscle. A total of 20,553 protein-coding genes, 7,615 known lncRNAs, and 196 novel lincRNAs were obtained. We compared the known and novel lincRNAs with the donkey protein-coding genes and found that known and novel lincRNAs have shorter transcript lengths, longer exon lengths, and fewer exon numbers compared with protein-coding transcripts, which is consistent with some previous studies (18–20, 27, 34–36, 43–46).

Differential expression analysis identified 272 DEGs, with 147 genes upregulated and 125 genes downregulated in the high-fat group compared to the low-fat group. Some of these genes may play a key role in lipid metabolism. As expected, GO analysis revealed the involvement of a significant number of DEGs in lipid metabolism-related biological processes, including response to lipid, cellular response to lipid, glycerophospholipid metabolic process, glycerolipid metabolic process, unsaturated fatty acid biosynthetic process, and phospholipid dephosphorylation. KEGG analysis showed significant enrichment of 62 KEGG pathways in the DEGs. Enriched pathways included the sphingolipid signaling pathway, MAPK signaling pathway, fatty acid metabolism, glycerophospholipid metabolism, biosynthesis of unsaturated fatty acids, PI3K-Akt signaling pathway, fatty acid degradation, ether lipid metabolism, cholesterol metabolism, PPAR signaling pathway, TGF-beta signaling pathway, and Wnt signaling pathway, which are involved in lipid metabolism.

Previous studies indicated that lncRNAs can regulate gene expression in a cis-acting manner (18, 20, 29, 35, 41). In the present study, a total of 196 novel lincRNAs and 7,615 known lncRNAs, were identified. Moreover, 52 DELs were detected, 25 of which were upregulated and 27 downregulated in the high-fat group compared with the low-fat group. To predict the function of these lncRNAs, protein-coding genes transcribed near lncRNAs (<100 kb) were screened. A total of 323 PTGs were identified, corresponding to 52 DELs. Exploration of lncRNA function through GO and KEGG analysis of PTGs revealed that 102 of 323 PTGs were significantly involved in 143 biological processes, and 59 PTGs significantly participated in 7 pathways, including 2 pathways related to lipid metabolism, such as biosynthesis of unsaturated fatty acids and fatty acid metabolism. In addition, four other pathways are related to lipid metabolism, namely the Wnt signaling pathway, glycerolipid metabolism, PPAR signaling pathway, and glycerophospholipid metabolism. These pathways can affect lipid metabolism to some extent.

An intriguing observation is that the same DEL ENSEAST00005078264 acts on both FADS1 and FADS2, which are the key enzymes that catalyze adenylation of flavin mononucleotide (FMN) to form flavin adenine dinucleotide (FAD) coenzyme (47, 48). These two PTGs are involved in the biosynthesis of unsaturated fatty acids and fatty acid metabolism. The DEL ENSEAST00005078264 may be involved in lipid metabolism via biosynthesis of unsaturated fatty acids and fatty acid metabolism signaling pathway. Another DEL ENSEAST00005069204 was found to target AGPAT5, which converts lysophosphatidic acid to phosphatidic acid, the second step in *de novo* phospholipid biosynthesis (49). These lncRNAs may regulate target genes involved in lipid metabolism pathways through cis-acting mechanisms.

The analysis of PTGs by trans-acting lncRNAs was conducted, and enriched analysis was also performed. Among the 3,366 PTGs, 1,352 were significantly involved in 835 biological processes. Pathway analysis showed that 710 PTGs were significantly involved in 74 pathways, including MAPK signaling pathway, PI3K-Akt signaling pathway, glycerolipid metabolism, ether lipid metabolism, fat digestion, and absorption, all of which are related to lipid metabolism. Other pathways related to lipid metabolisms, such as glycerophospholipid metabolism, fatty acid metabolism, biosynthesis of unsaturated fatty acids, sphingolipid signaling pathway, cholesterol metabolism, adipocytokine signaling pathway, Wnt signaling pathway, fatty acid elongation, regulation of lipolysis in adipocytes, sphingolipid metabolism, TGF-beta signaling pathway, fatty acid biosynthesis, and fatty acid degradation, were also identified. For 132 DEPTG, 56 were significantly involved in 143 biological processes. Pathway analysis showed that 27 DEPTGs were significantly involved in 7 pathways, including arachidonic acid metabolism. Other pathways involved in lipid metabolism have also been identified, including biosynthesis of unsaturated fatty acids, fat digestion and absorption, ether lipid metabolism, fatty acid metabolism, MAPK signaling pathway, PPAR signaling pathway, glycerophospholipid metabolism, and PI3K-Akt signaling pathway.

It was found that SCD, targeted by four DELs (ENSEAST00005042127, ENSEAST00005051768, ENSEAST00005052324, and ENSEAST00005072263), is a key gene that regulates lipid metabolism. Studies have shown that SCD plays an important role in lipid biosynthesis (50–52). In addition, two DELs, ENSEAST00005052438 and ENSEAST00005076477, were all found to act on PLA2G3, which is involved in lipid metabolism and catalyzes the calcium-dependent hydrolysis of the sn-2 acyl bond of phospholipids to release arachidonic acid and lysophospholipids (53–56). It is noteworthy that THRSP was targeted by four DELs (ENSEAST00005042127, ENSEAST00005048792, ENSEAST00005051768, and ENSEAST00005052324). Previous studies showed that THRSP is important for the biosynthesis of triglycerides with medium-length fatty acid chains and plays a role in the regulation of lipogenesis, which may modulate lipogenesis by interacting with MID1IP1 and preventing its interaction with ACACA, may function as a transcriptional coactivator and may modulate the transcription factor activity of THRB (57, 58). These lncRNAs may regulate target genes involved in lipid metabolism pathways through trans-acting mechanisms.

SCD plays an important role in the regulation of lipid deposition, and it can catalyze the conversion of saturated fatty acids (SFAs) to monounsaturated fatty acids (MUFAs) (59). It was found that SCD expression was positively correlated with SCD protein levels, and pigs with higher SCD protein levels had higher levels of IMF, indicating a conserved trend between transcriptional and translational levels (60). Smith et al. (61) found that SCD expression was closely related to marbled adipocyte differentiation and that grain diets increased SCD expression, resulting in higher intracellular levels of MUFA (especially oleic acid), and thus increased adipocyte differentiation. In addition, increased FA levels in IMF tissues were found to be associated with tissue-specific activation of SCD expression under the influence of a low-protein protein diet, and the low-protein protein diet significantly increased the expression and activity of SCD proteins in muscle, but not in subcutaneous adipose tissue (AT) (62). These results suggest that the high specific expression of SCD in IMF cells may be related to meat quality and that reduced protein intake may affect IMF content by modulating SCD activity. Notably, SCD was significantly up-regulated in the high-fat group compared with the low-fat group in this study. The results show that SCD may play an important function in donkey IMF deposition. The thyroid hormone-sensitive protein (THRSP; Spot14; S14) is a nuclear protein that is abundantly expressed in lipogenic tissues such as in the liver, mammary gland, AT and lipogenic breast cancers (63–66). A previous study indicated that the polymorphisms and genotype distribution of THRSP were closely related to the potential for fat production in pig breeds (67). It was found that miR-195 may inhibit lipid accumulation in adipocytes by regulating THRSP (68). THRSP is regulated by insulin both *in vivo* in human AT and *in vitro* in adipocytes, and its expression is downregulated by insulin resistance. As THRSP silencing decreases mitochondrial respiration and fatty acid oxidation, its downregulation in human AT could contribute to mitochondrial dysfunction. Furthermore, disturbed sphingolipid metabolism could contribute to metabolic dysfunction in obese AT (69). The current RNA-seq results revealed that THRSP had higher expression in the high-fat group than in the low-fat group, which suggested that THRSP may be a key gene involved in IMF deposition in donkeys. Taken together, some DEGs, including SCD and THRSP, may be key candidate genes for donkey meat quality improvement.

It is noteworthy that SCD was targeted by four DELs (ENSEAST00005042127, ENSEAST00005051768, ENSEAST00005052324, and ENSEAST00005072263). Moreover, four DELs (ENSEAST00005042127, ENSEAST00005048792, ENSEAST00005051768, and ENSEAST00005052324) were found to target THRSP. These lncRNAs may influence donkey IMF deposition by regulating the expression of their target genes. The relationship of these lncRNAs with their target genes and with fat deposition in donkey muscle needs to be further investigated in the future using molecular biology, gene editing, etc. These lncRNAs may improve meat quality and facilitate the selection process of donkeys in future breeding.

## 5 Conclusion

In the study, we identified 196 putative lncRNAs and analyzed the characteristics of lncRNAs compared with protein-coding genes in the longissimus dorsi muscles of Guangling donkeys. We observed numerous DELs and protein-coding genes in longissimus dorsi muscles with different IMF contents. Some DEGs were found to be involved in various biological processes related to lipid metabolism. Functional enrichment analysis of PTGs by DELs revealed that some lncRNAs (such as ENSEAST00005042127, ENSEAST00005051768, ENSEAST00005052324, ENSEAST00005072263, ENSEAST00005052438, ENSEAST00005076477, ENSEAST00005042127, ENSEAST00005048792, ENSEAST00005051768, and ENSEAST00005052324) may act on PTGs (such as SCD, PLA2G3, and THRSP), participate in lipid metabolism processes, and regulate IMF deposition in the longissimus dorsi muscle. This study provides valuable resources for future analyses of lipid deposition traits and may contribute to the improvement of donkey meat quality and the selection process in donkey breeding.

## Data availability statement

The datasets presented in this study can be found in online repositories. The names of the repository/repositories and accession number(s) can be found in the article/Supplementary material.

## Ethics statement

The animal studies were approved by Animal Ethics Committee of Liaocheng University. The studies were conducted in accordance with the local legislation and institutional requirements. Written informed consent was obtained from the owners for the participation of their animals in this study.

## Author contributions

YP: Conceptualization, Data curation, Formal analysis, Funding acquisition, Investigation, Methodology, Software, Visualization, Writing – original draft, Writing – review & editing. MZ: Data curation, Investigation, Methodology, Visualization, Writing – review & editing. YG: Data curation, Formal analysis, Methodology, Software, Writing – review & editing. CW: Conceptualization, Funding acquisition, Project administration, Resources, Supervision, Validation, Visualization, Writing – original draft, Writing – review & editing.

## Footnotes

^1^ http://kobas.cbi.pku.edu.cn
